# The Next Horizon of Drug Development: External Control Arms and Innovative Tools to Enrich Clinical Trial Data

**DOI:** 10.1007/s43441-024-00627-4

**Published:** 2024-03-25

**Authors:** Kelly H. Zou, Chelsea Vigna, Aniketh Talwai, Rahul Jain, Aaron Galaznik, Marc L. Berger, Jim Z. Li

**Affiliations:** 1 Viatris, Canonsburg, PA USA; 2 Medidata Solutions, a Dassault Systèmes Company, Boston, MA USA

**Keywords:** Clinical trial, External control arm, Decentralized clinical trials, Real-world data, Real-world evidence, Record linkage, Patient diversity

## Abstract

Conducting clinical trials (CTs) has become increasingly costly and complex in terms of designing and operationalizing. These challenges exist in running CTs on novel therapies, particularly in oncology and rare diseases, where CTs increasingly target narrower patient groups. In this study, we describe external control arms (ECA) and other relevant tools, such as virtualization and decentralized clinical trials (DCTs), and the ability to follow the clinical trial subjects in the real world using tokenization. ECAs are typically constructed by identifying appropriate external sources of data, then by cleaning and standardizing it to create an analysis-ready data file, and finally, by matching subjects in the external data with the subjects in the CT of interest. In addition, ECA tools also include subject-level meta-analysis and simulated subjects’ data for analyses. By implementing the recent advances in digital health technologies and devices, virtualization, and DCTs, realigning of CTs from site-centric designs to virtual, decentralized, and patient-centric designs can be done, which reduces the patient burden to participate in the CTs and encourages diversity. Tokenization technology allows linking the CT data with real-world data (RWD), creating more comprehensive and longitudinal outcome measures. These tools provide robust ways to enrich the CT data for informed decision-making, reduce the burden on subjects and costs of trial operations, and augment the insights gained for the CT data.

## Introduction

Successful execution of clinical trials (CTs) has always been a significant challenge for drug development [1]. Over the past decade, the magnitude and impact of clinical trials have grown significantly due to their complexity, multifaceted design, and challenging operational requirements (e.g., complex tissue sampling, and molecular and processing requirements). In addition, the execution of these complex CTs is further compounded by the difficulty in finding suitable study sites [2, 3], staff turnover, especially in academic centers where there is high competition from industry [1], loss to follow-up of the study subjects, enrollment of appropriate patient population, and, more recently, COVID-19 pandemic restrictions and challenges [1].

In this article, we will review some robust sets of tools that can help the industry overcome some of these challenges; predominant among these is the incorporation of additional data from external control arms (ECAs) and other pooled subject-level data to complement or supplement data collected from traditional CTs [4–13]. Other novel tools of specific interest include innovations associated with the nature and conduct of clinical trials themselves (such as the virtualization of RCTs [14, 15]) and the ability to follow the clinical trial subjects in the real world by linking the two disparate sources of health data using tokenization [5, 16].

## External Control Arms

### Definitions, Categories, and Construction

The International Council for Harmonisation of Technical Requirements for Pharmaceuticals for Human Use (ICH) Guideline E10 defines an externally controlled trial as “one in which the control group consists of subjects who are not part of the same randomized study as the group receiving the investigational agent, i.e., there is no concurrently randomized control group” [17]. While external controls are utilized as stand-alone comparators, a trial may also enroll subjects in a concurrent control arm and augment it using an external control arm (hybrid control arms) [18, 19]. Recently, the U.S. Food & Drug Administration has issued a draft guidance on externally controlled trials [6].

ECAs may be categorized as concurrent ECAs and historical ECAs: concurrent ECAs use subject data collected during the same time-periods as the subjects receiving the investigational agents, while historical ECAs use subject data collected at an earlier time [17]. An ECA is constructed by choosing the control arm’s subjects for comparison with current experimentally treated subjects and usually comprises the following steps [7]:

1. Identification of an external data source that provides a similar population as studied in the clinical trial.
2. Data entry, reading, and processing to generate an analysis-ready data file.
3. Statistical selection or adjustments using subject-level data to balance baseline covariates between arms.

### Data Sources

While data for concurrent ECAs typically come from concurrent observational studies or concurrent patient registries, historical ECAs may use data from historical CTs or real-world data (RWD) such as historical observational studies, patient registries, electronic health records, insurance claims, or publications.

Historical CT data, especially those from large and well-conducted RCTs for the same disease and similar patient populations, are more suitable when the RCTs follow ICH guidelines. Such data usually are more accurate and complete than most RWD, generally have baseline demographic and clinical characteristics variables similar to those for the target clinical trial and are more likely to use similar definitions for disease, patient inclusion and exclusion criteria, and outcome measures. Historical CT data are often used when pooled RCT data are available. Examples of sources for the pooled subject-level clinical trial data include the Historical Trial Data (HTD) Sharing Initiative [20] and Medidata Enterprise Data Store (MEDS) [5]. HTD Sharing Initiative was established to share de-identified data to maximize the value of clinical data collected historically in the control arms of clinical trials [20]. The MEDS has amassed a pool of more than six million anonymized subjects from nearly 20,000 previous clinical trials [4]. Other sources include Project Data Sphere (PDS), which collects, curates, and aggregates clinical trial data on its open-access platform allowing researchers to develop external control arms from subject-level data [21]. The Yale University Open Data Access (YODA) Project is another source of open-source data access [22].

RWD may also be leveraged to create external controls [9, 13, 23–26]. RWD is a particularly useful data source when historical RCT data are unavailable or unsuitable for comparison, e.g., in rare diseases where there is often a paucity of prior CTs due to a lack of available treatments and insufficient sample sizes in patient enrollment. The frameworks for RWD and real-world evidence (RWE) have been developed by the United States (US) Food and Drug Administration (FDA), the EMA of the European Union (EU), and Japan’s Pharmaceuticals and Medical Devices Agency (PMDA). These regulatory agencies support the various uses of RWD for regulatory purposes [27–30]. Because the collection of RWD often does not follow ICH guidelines or clinical practices, researchers need to closely examine the validity, reliability, and relevancy of the data when using them to create ECAs.

Irrespective of the data source, the quality of the ECA depends on the comparability of treatment approaches, completeness of patient attributes captured, and the robustness of endpoint assessment to ensure good matching using methods such as propensity scores and comparability with experimental trial subjects.

### Applications

ECAs are particularly useful where implementing an RCT may not be feasible or ethical. These may include, for example, testing an investigational drug for a rare disease with no alternative treatment or established standard of care, or subjects are very difficult to find for a disease with high unmet need (e.g., a fast progression cancer with increased mortality, or for a vulnerable population such as pediatric subjects.) [23–25]. For indications where CTs are often operated as single-arm trials where all participating subjects are assigned the investigational drug [8], ECAs may provide the data needed to assess the efficacy and safety of the investigational intervention as seen for example in Celsion’ OVATION trials using Medidata Synthetic Control Arm® [31].

For RCTs testing drugs for conditions with an inadequate standard of care, hybrid ECA designs have been suggested to augment in-trial control arms. In this approach, multiple subjects are included in the external control arm for each subject in the control arm, i.e., at a *k*:1 ratio. This hybrid approach allows more subjects to be randomized to the investigational drug while preserving some randomizations [4, 19, 32].

### Benefits

The use of ECAs allows the entire or a larger proportion of the participants of a CT to be assigned to the experimental treatment arm, which significantly boosts patient welfare when the novel treatment is hypothesized to have better safety or efficacy compared to the standard of care. This is particularly important when no current treatment exists. This advantage also obviates the quandary when subjects do not want to be assigned to a standard of care that they may perceive inadequate. Not only ECAs allow a larger proportion of patients to be assigned to the investigational arm, but it also ensures that the quality of evidence generated by CTs in diseases with small and/or hard-to-recruit populations is high and helps enhance the inclusion of such populations.

Improved trial efficiency could allow RCTs to complete faster, enabling drugs to get approval and market faster (if the ECA methods used and trial results are accepted by regulators)—thereby benefitting subjects not enrolled in CTs who might otherwise have inferior (or no) treatment options. Besides shortening the time for new drug approval and time to market, improving trial efficiency also helps reduce the cost to sponsors for new drug development by reducing the number of subjects needed for the CTs required for the drug’s approval. There are several benefits in terms of metrics [33].

In addition, it has been suggested that ECAs may also provide sponsors and regulators in the future with the evidence needed to support expedited conditional approval or with an additional source of evidence to translate conditional approvals to full approvals or approve additional indications (label expansion), increasing the pool of subjects who can benefit from the therapy [24]. ECA may also allow for the comparison of the investigational drug against a broader set of comparators and patient types.

In cases where the comparator arm may have otherwise been compromised (e.g., due to lower adherence or higher dropout rates if the comparator treatment becomes less effective due to evolutions in clinical practice over the course of the CT [34]), a carefully selected ECA cohort can still help estimate the treatment effect with a high degree of accuracy. ECAs can also help when subjects may be reluctant to enroll if the comparator or reference product has been superseded in clinical practice or there is a perceived risk–benefit tradeoff with older products (e.g., nocebo effect) [35].

### Challenges and Potential Biases

The biggest challenge is to find relevant and high-quality data for ECAs, as discussed above. As more and more sponsors have contributed their historical RCT data to the pooled CT databases such as the Historical Trial Data (HTD) Sharing Initiative and the Medidata Enterprise Data Store (MEDS) mentioned above, and as more and more RWD become available, the shortage of relevant and high-quality data for ECAs can be gradually eased.

Another major challenge is the potential confounder and biases, especially for RWD-based ECAs, which can make it difficult to estimate with confidence the efficacy and safety profile of the investigational therapy [36, 37]. A confounder is a variable correlated with both the outcome and the intervention without being an intermediate cause in the causal pathway between intervention and outcome. It is essential to find and use data with a sufficiently large number of covariates/baseline variables to identify the potential confounders and minimize the potential biases [36–39].

Without the needed variables, no statistical methods may be able to comprehensively correct for all potential confounding factors that have been identified by other researchers in other studies. When the appropriate data are available, advanced statistical methods may be used to reduce or remedy the potential biases caused by those confounding factors. These methods are discussed in more detail in the sections below.

As with other external controls, the nature and quality of the underlying external data are critical for the rigor and validity of ECAs. Thus, several biases may affect these data sources, and statistical methods may be considered to mitigate their effects.

One of the main reasons regulatory agencies favor randomization in CTs, i.e., randomized controlled trials (RCTs), is to clearly establish a potential causal link between a therapy and the observed outcome [40–44]. These approaches can account for effects of treatment intent, time-varying treatment, and confounding for multiple treatment effects [45]. RCT emulations may also be conducted [16, 46, 47], but due to a lack of randomization. However, there are potential biases for consideration when building an ECA (Table 1).

**Table 1:** Sources of Biases [6, 74, 117, 120]

Number	Type of bias	Description
1	Selection	Regardless of the data source, the external validity of results will be affected if patient characteristics greatly vary between the ECA and the investigational treatment arm [20]
2	Confounding	This is especially important to consider when building an ECA using RWD since specific patient characteristics are also likely to influence the treatment that they are given. For example, patients that are sicker may be given more aggressive treatment and may also experience worse outcomes. Confounding and other biases can also be present due to the differences in sites. For example, there can be differences in care protocols, the volume of patients seen at sites, and the type of the care setting—academic versus community [24]
3	Ascertainment	When the frequency and rigor of outcome assessments differ between CTs and external control, this presents a risk of comparability and compatibility. If the frequency and intensity of clinical interaction differ across groups, it impacts the likelihood of detection of trial outcomes and/or adverse events and impacts endpoints (e.g., via time-to-event analysis). Patients assessed less frequently will, by extension, have a longer time in general to detection of certain clinical outcomes and hence, longer time-to-event durations. In RWD, the frequency of assessment will be dictated by the frequency at which a patient *chooses* to interact with the healthcare system. As such, there will be more variability and likely longer time intervals between assessments that must be accounted for when making clinical trial-to-real-world comparisons [24]
4	Compliance/adherence to treatment regimens	While less applicable to ECAs derived from historical trial data, this is a factor that should be considered and incorporated when RWD is used. Non-adherent patients would be expected to be biased to worse outcomes compared to adherent patients. Compliance bias may not solely be a behavioral issue but a structural one in how patients interact with the healthcare system. Certain social determinants of health may also affect a patient’s adherence, such as access to transportation to healthcare centers
5	Type 1 error	The literature on Bayesian basket trials (where strength is borrowed across similar but not identical baskets) offers a calibrated Bayesian approach whose goal is to reduce the risk of unacceptably high Type 1 error when borrowing turns out to be unwarranted

### Matching Methods

Advanced methods (e.g., propensity score matching [PSM]) are increasingly applied to ensure that the subjects in the current trial and historical benchmarks are as similar as possible. Reducing the differences between the patient characteristics in an experimental arm and an ECA can be achieved through matching methods, which also address sources of confounding and selection bias. Confounding was discussed earlier, while selection bias is best described as a “fundamental difference between the patients included in the treatment arms of a study due to the way in which patients were allocated to the treatment groups” [48].

Some recent case examples of ECAs that have employed PSM include those from the Friends of Cancer Research working group in both Lung Cancer and Multiple Myeloma [8, 49]. PSM of pooled subject-level historical trial data was used to replicate results from the control groups of prior CTs with a high degree of similarity to the original outcomes. Additionally, regulatory guidance documents suggest that reducing selection bias starts with a priori selection of the external control group before conducting any comparative analyses and suggests documenting the analytic approaches in a pre-specified protocol and statistical analysis plan [26].

### Bayesian Methods

Bayesian approaches have been applied to CTs for adaptive data borrowing, including power priors, commensurate priors, meta-analytic predictive priors, and robust mixture priors [50–53]. For example, the Bayesian case example repository, supported by the Drug Information Association’s Bayesian Scientific Working Group, contains a series of case studies demonstrating examples of the use and value of Bayesian statistics in medical product development [54]. In particular, it can be useful for pediatric trial designs [55]. Additionally, the FDA recognizes and provides guidance on Bayesian adaptive designs [56, 57]. However, it is worth noting that the FDA cautions about using adaptive designs with smaller sample sizes, as they may fail to provide outcomes on subpopulations with insufficient statistical power [56]. This is particularly pertinent for hybrid study designs with small samples, where historical information can be used to inform prior distribution, increasing the statistical power for future (i.e., posterior) conclusions [58]. These approaches are readily applicable to external controls [59].

### Timing of Trials

It is essential to account for the differences in timing to capture study observations between a CT and external control. This pertains to mitigating sources of ascertainment bias. Here, ascertainment bias is “the systematic distortion of the assessment of outcome measures by researchers or study participants” [60].

Using a historical CT for ECAs mitigates much of this concern in ascertainment bias, as external data are likely to be from a similar setting of control and scrutiny. However, care should still be taken to review trial protocols and assess the similarity of periodicity and rigor of assessment in trial data included [8]. Careful selection of matching variables and matching approaches should be used. Another method for identifying and adjusting for ascertainment bias is using positive and negative controls, where positive controls are the variables known to impact the outcomes of interest and negative controls are variables that are known not to causally affect the outcome [61].

In a study by Desai et al., an association of diabetes with both hereditary fructose intolerance and Alpha-1 Antitrypsin deficiency, two rare diseases, was assessed across multiple data sources [62]. Positive and negative controls were used to calibrate the strength of association to account for possibly higher levels of examination and intervention in diagnosed rare disease subjects. A similar approach was used by Schuemie et al. in RWD to compare associations with dabigatran, warfarin, and gastrointestinal bleeding, as well as those of selective serotonin reuptake inhibitors and upper gastrointestinal bleeding [63]. Both examples used positive and negative controls to calibrate confidence intervals to determine the statistical significance of observed effect sizes. The discrepancies between two conflicting RWE studies were explained [64]. Addressing the treatment adherence/compliance bias requires active awareness of this issue and ensuring adequate insight into the data to assess it. Consideration of screen failure rates and discontinuation rates is required for historical RCTs. For RWD, sufficient capture of diagnosis, healthcare encounters, procedures, treatment administration, and prescription fill or refills, etc., as pertinent to the question at hand is required.

### Subject Level Meta-Analysis

Pooling of historical CT data and/or RWD also enables various other applications, including target selection for new mechanisms, trial design and optimization, trial recruitment, health technology assessment, and market access approval, and post-approval applications for the verification of effectiveness and life cycle management, label expansion, and drug repurposing [65].

A meta-analysis should be conducted to estimate the treatment effect associated with the intervention and to understand the uncertainty around the effect. Traditional meta-analyses use aggregate results from multiple CTs based on data available in publications or on an individual patient level. In clinical development, they often serve as a starting point for effect size estimates in trial planning, aiding in comparator selection and power calculations. Meta-analyses can be of aggregated data reported in the studies or of the individual subject-level data. Data can then be systematically pooled (e.g., random-effects model or fixed-effects model [66, 67]), affording a greater sample size than can be achieved. Within pooled data, inclusion and exclusion criteria can be matched towards a potential new CT. Multi-arm (e.g., indications and dosages) trial cohorts can be stratified as needed. The timing of outcome assessments measured can be aligned for consistency. For differences in composite endpoint calculation, individual outcome elements, if available, can be used to standardize outcome assessments across trials.

### Lifecycle Management

An important component of extending the value of therapeutics is lifecycle management (LCM). This can include maintaining market approval, as well as enhancement of value through indication expansion, reformulation, or repurposing [68]. Maintaining market approval is an issue that has arisen in Europe, the Middle East, and Africa for long-approved off-patent products in disease areas where newer (potentially more efficacious/effective) treatments are available, and standard of care has evolved. In such cases, the regulators may seek the assurance of continued therapeutic benefit as part of market re-authorization, and the lifecycle stage may not be conducive to conducting Phase IV trials. For these cases, Pooled CT data for external comparators have several advantages over RWD alone. When paired with subject-level meta-analytic or ECA approaches, this provides the ability to compare evolving performance benchmarks over time, although such comparisons may be limited or infeasible if diagnostic criteria or endpoint preferences have significantly changed. With the advent of interchangeable biosimilars (a biosimilar product that may be substituted without the intervention of the healthcare professional who prescribed the reference product, much like a generic drug for a branded drug) [69, 70] in the US, the above approach also has potential applications for supporting future biosimilar approvals [71]. Indication expansion and drug repurposing efforts may similarly benefit from an external benchmark, ECA, or hybrid approaches. As the drugs in question have already met efficacy and safety hurdles, there is already a precedent for the supportive use of RWE in this application through existing and ongoing RWD and RWE. The FDA approval of palbociclib for male breast cancer, which was expanded from female breast cancer, included supportive EHR data (see, e.g., [72]). When paired with ECA or hybrid approaches, accelerated drug development and approval may possibly be achieved.

### Simulated Data

A promising and emerging approach for working with subject-level data in a secure manner is to employ simulated data. Simulated subject-level data can be created from existing data to preserve patient anonymity and prevent accidental or potential identification of subjects. This can be applied to either RWD or CT data. Simulated data preserve the relationships that exist in source data, but they alter the identifying information about each of the subjects that make up the cohort. Unlike individual-level meta-analyses, simulated data may more easily be shared without patient-specific information.

By allowing the use of historical CT data while preserving patient anonymity, a full anonymization or de-identification approach increases the flexibility in leveraging these databases. While the terms anonymization and de-identification may be considered synonymous terms, there are some subtle different meanings between them and regulatory preferences of the two terms [73–75].

According to EDUCAUSE (https://www.educause.edu/), anonymization is “the act of permanently and completely removing personal identifiers from data, such as converting personally identifiable information into aggregated data. Anonymized data is data that can no longer be associated with an individual in any manner.” In comparison, “de-identification involves the removal of personally identifying information in order to protect personal privacy. In some definitions, de-identified data may not necessarily be anonymized data. This may mean that the personally identifying information may be able to be re-associated with the data at a later time” [76]. Europe’s General Data Protection Regulation (GDPR) tends to use the term anonymization and defines anonymous information as the “information which does not relate to an identified or identifiable natural person or to personal data rendered anonymous in such a manner that the data subject is not or no longer identifiable.” (GDPR Recital 26) [77]. In comparison, the US regulations tend to use the term de-identification. For example, the Health Insurance Portability and Accountability Act (HIPAA) defines de-identification as the process by which identifiers are removed from the health information following the de-identification standard and implementation specifications in HIPPA §164.514(a)-(b), and the de-identified health information as the “health information that does not identify an individual and with respect to which there is no reasonable basis to believe that the information can be used to identify an individual.” (HIPPA §164.514) [78]. The California Consumer Privacy Act (CCPA) defines de-identified information as “information that cannot reasonably identify, relate to, describe, be capable of being associated with, or be linked, directly or indirectly, to a particular consumer” [79].

Simulated data have been evaluated in both CTs and canonical non-CT (e.g., handwriting, news topic) datasets for both fidelity (i.e., how well statistical relationships are maintained) and privacy (i.e., how well the identity and private information of sponsors or trial participants can be maintained) and have demonstrated equal or superior performance when benchmarked against other state-of-the-art data generators.

Simulations have the potential to broaden CT, allowing sponsors with fewer of their own CTs to access extensive clinical trial datasets long before producing their own dataset. On specific aspects of ECAs, please see Table 1 of the United States Food & Drug Administration’s Guidance on ECA [6]. Simulation would usually consist of generating a synthetic dataset in the same format and structure as the original source data (e.g., ADaM, SDTM, etc.), which would collectively retain the same statistical properties as the original (so that any clinical relationships & insights are preserved), while ensuring that no individual’s patient data from the source can be derived from the synthetic [73, 80].

Simulations may also be used to gain early insights into outcomes for the control arm. Emerging insights from the early applications of this approach (e.g., Medidata Simulants approach [73]) have also been validated from interim results of the ongoing CT. Advancing the methodology in retaining the relationships of adverse events from the source data to simulated data is ongoing. In addition to the expansion of data available to investigators, innovations in technology have enabled the structure and conduct of clinical trials themselves [81].

Protecting subjects’ privacy is an important aspect, and protected health information (PHI) under “the US HIPAA Privacy Rule provides federal protections for personal health information held by covered entities and gives patients an array of rights with respect to that information. At the same time, the Privacy Rule is balanced so that it permits the disclosure of personal health information needed for patient care and other important purposes” [82].

For example, according to the U.S. National Institute of Standards and Technology (NIST), anonymization is the “process that removes the association between the identifying dataset and the data subject” [83]. In contrast, “de-identification is a way for organizations to remove personal information from data that they collect, use, archive, and share with other organizations.” It is worth noting that “de-identification is not a single technique, but a collection of approaches, algorithms, and tools that can be applied to different kinds of data with differing levels of effectiveness” [83]. See US Census bureau about privacy and methods for preserving anonymity and use of methods such as “differential privacy” for protecting privacy [84].

### Regulatory Perspectives

In general, regulators, including FDA and EMA, support the use of external controls for regulatory purpose but requires the investigators to show the appropriateness of the data and methodologies used for external controls [85]. Jahanshahi et al. reviewed FDA regulatory approval decisions between 2000 and 2019 for drug and biologic products to identify pivotal studies that leveraged external controls. They identified 45 approvals where FDA accepted external control data in their benefit/risk assessment; they did so for many reasons, including the rare nature of the disease, ethical concerns regarding the use of a placebo or no-treatment arm, the seriousness of the condition, and the high unmet medical need [9]. In another review article, Goring et al. identified applications of 43 products submitted to FDA (n = 41) or EMA (n = 34) between 2005 and 2017 that used non-randomized studies using comparisons with external controls. They found that FDA approved 98% of submissions, with 56% accelerated approvals; most required post-approval confirmatory randomized controlled trials (RCT); EMA approved 79% of submissions, with a quarter of approvals conditional on completion of a post-approval RCT or additional non-randomized trials [8]. Throughout the remaining of this article, we focus on new drug development, for medical devices, diagnostics or new vaccines, as well as value added medicines (VAMs) that contribute to addressing unmet patient needs [86].

These favorable opinions by FDA are seen in cases where the disease has high and predictable mortality. In other words, the diseases are well understood, with objective endpoints, and the effects of baseline patient and treatment characteristics on endpoints are well characterized. Detailed subject-level information needs to be available, typically in 100 s of variables, including demographics, comorbid conditions, therapies, concomitant medications, and others.

Regulators, while they have favorable opinions on the use of ECAs in general, have also cautioned against the overuse of ECAs. The burden of addressing potential confounders is also heavier on the ECAs than on traditional CTs. In general, the conventional practice is to involve regulators as early as possible to mitigate future regulatory risks [87]. In a recently published article by FDA officers, they shared their thoughts on the future directions and considerations for ECAs [87].

## Other Relevant Tools

### Decentralized Clinical Trials

CTs have been historically site-centric, as sites are the venue where all interactions between the investigators and the trial participants take place and are typically tasked with documenting the progress and associated CT conduct.

However, this site-centric approach imposes limitations and challenges for both participants and investigators, including the following:

- The travel burden on trial participants is likely to restrict the trial population to those who live in geographic proximity to the clinical site, leaving many subjects excluded, affecting the generalizability of the trial results [87, 88].
- Only static and periodic measures may be collected and obtained in specific clinical settings, such as a hospital, clinic, and long-term care facility [81].
- Inability to collect data when the patient is off-site (i.e., for the vast majority of the clinical trial’s duration) and in their natural routine potentially affecting real-life adherence and applicability of therapies [88].

However, with advances in digital health technologies, CT conduct is slowly shifting away from this site-centric design to decentralized clinical trials (DCTs) [89].

**Figure 1:**
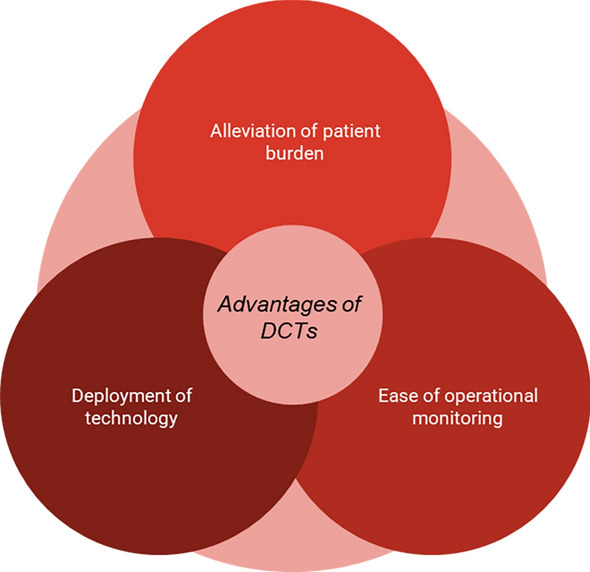
Advantages of decentralized clinical trials.

Virtualizing a CT helps alleviate current challenges and issues experienced in traditional clinical trials, including recruiting and obtaining consent from each subject into a study (Fig. 1). While recruitment is a key measure of a study’s success, it remains an ongoing and complex challenge [90]. As many as 86% of trials in the US [90], 69% of trials in the United Kingdom (UK) [92], and over 90% in Australia [91] do not hit their target enrollments which causes delays. According to Fogel [92] “There are many reasons that potentially efficacious drugs can still fail to demonstrate efficacy, including a flawed study design, an inappropriate statistical endpoint, or simply having an underpowered clinical trial (i.e., sample size too small to reject the null hypothesis), which may result from patient dropouts and insufficient enrollment.”

Even remuneration to compensate subjects for their time and travel burden has not shown conclusive impacts on driving enrollment [91–93]. These delays not only adversely affect the investigators and sponsors but also lengthen the time to market of often desperately needed therapies. Some of the barriers to low recruitment are a lack of access to eligible subjects, a lack of subjects’ understanding of the study protocol, and unclear or uncertain burden [89]—each of which can be addressed, at least in part, by virtualization of the clinical trial.

The expanded use of personal electronic devices, such as smartphones, has allowed continuous, real-time, two-way communication between investigators and trial participants. In addition to patient convenience, these bring your own device (BYOD) design methods also bring potential for observing and collecting data domains or measures that were not possible previously [94]. Such methods may also be subject to patient-centric considerations and complexities, such as patient-reported outcomes and data privacies [95]. Such patient-centric considerations, especially in an era of artificial intelligence for drug development and other purposes, are increasingly seen [96–101]. These technological innovations can be adopted to maintain high levels of engagement, such as digital health [94] and artificial intelligence [96], among the trial participants through interactions that can respond to participants’ queries, prompt adherence to prescribed protocols, and incorporate participant feedback to fit the CT into their daily life [102].

One aspect of trial virtualization is through centralized, often cloud-based, platforms to facilitate data gathering, monitoring, and outreach across sites. According to the FDA, “foundational requirements for a modern FDA technology infrastructure include virtual data storage (“the cloud”), problem-specific software” [103]. Consequently, there is an opportunity for virtual site monitoring and real-time gathering of CT performance metrics [104]. Virtual site monitoring reduces the need for travel, thereby alleviating the burden both on sites and contract research organizations. Centralized, real-time monitoring of trial enrollment also allows for rapid identification of challenges at the site level and the opportunity for rapid intervention [105]. Recent work has shown how this type of data can be used to predict trial dropout [106]. Furthermore, with centralized data collection, statistical algorithms can be deployed to detect erroneous or anomalous data entries, while preserving subject privacy and de-identification, enabling rapid corrective actions and site education [107].

To varying degrees, DCTs have already seen acceptance by various regulators [107] and in subject matters such as oncology [108]. However, the level of virtualization of trials should be need-specific by balancing risks against operational necessities and designed early in the protocol development while working with regulators. [107] Risk-assessment tools can be utilized to determine the overall risk associated with the specific trial design in mind [107]. Potential barriers to the adoption of DCTs are the upfront load on the site clinicians involved in the initial set-up process and the fit-for-purpose PRO instruments [109]. However, collaborations are critical among health research organizations, health systems, and other stakeholders [110].

### Data Linkage at the Patient Level

According to the FDA’s guidance, “data linkages can be used to increase the breadth and depth of data on individual patients over time and provide additional data for validation purposes” [111].

For example, sponsors are increasingly looking to the linkage of CT data and RWD at the patient level as the next frontier of clinical development. Combining seemingly disparate data sources may maximize the potential of complex CT data and the ever-growing repository of RWD generated by healthcare systems. With record linkage, it is possible to generate and accelerate key insights on healthcare resource utilization (HCRU), outcomes, and the intricacies of patient pathways throughout all phases of clinical development [111].

CT data and RWD have long existed in silos due to technological, privacy, and regulatory reasons. Such data chasms have prevented sponsors from painting a comprehensive patient journey and harnessing insights from multiple data sources and types. While barriers due to data fragmentation are significant, the linkage of CT data and RWD offers a holistic approach to understanding subjects through outcomes research.

To link RCT data with RWD at the patient level, patient identifiable information and informed consent forms must first be collected and stored in a highly secure environment. Tokenization is particularly useful to link data will ensure compliance with privacy protection regulations including the Health Insurance Portability and Accountability Act of 1996 (HIPAA), EU General Data Protection Regulation (GDPR), Institutional Review Boards (IRB), and Ethics Committees (EC) [112–115]. Tokenization is a process of de-identification by removing patient identifiers and generating patient-specific encrypted tokens [117]. Specifically, from a patient’s identifiers a de-identified and encrypted token is created which is used to replace the patient’s identifiers. The token can be used to determine which real-world datasets that a patient exists in. Sponsors may strategically select patient-specific RWD to combine with CT data. What results is a combined clinical and real-world dataset that can be augmented over time. These goals may be achieved via record linkage, including CT diversity and data representativeness. Such a comprehensive dataset allows the sponsor to follow the trial participant and describe the patient journey from CT and beyond.

Record linkage generates a complete view of a patient at different trial phases and enables the sponsor to track long-term safety, efficacy, and HCRU outcomes that may not be captured within a CT only. Record linkage must be done in a privacy-preserving manner. Specifically, pre-trial linked data can facilitate enhanced baseline data and information to minimize potential delays. Furthermore, during the CT conduct, record linkage enables a deeper and fuller understanding of total HCRU and potential behavior and rationales behind any loss to follow-up [116–118].

## Summary

We have presented an overview of several useful CT tools, which may facilitate trial comparators, provide external control subjects, enhance diversity in participating patients, and augment the insights gained for the CT data. While the successful execution of clinical trials is critical for drug development, its magnitude and impact have grown significantly over the past decade (also see [119]).

Notable recent advancements in CT conduct include virtualization of the clinical trial, tokenization and linking of CT data and RWD, and simulating patient data. The specific CT tools presented here include ECA, trial virtualization, and tokenization. These CT tools may improve patient diversity, sharpen the precision in outcome measures in CT, enrich clinical trial data, and provide alternative pathways for gathering evidence of efficacy. Finally, they provide robust ways to enrich the CT data and RWD for informed decision-making, reduce the burden on subjects and costs to trial operations, and increase the value of CT data [6]. According to the FDA [6], there are still potential limitations of the ECA approaches, including availability of data, comparability of data, missing data, misclassification of data, and most seriously, increased bias and Type I error [120]. Some of these limitations may be dealt with via careful statistical considerations. Others may remain challenging, however.

Finally, as the regulatory landscape continues to evolve and the volumes and complexities of data generated by our healthcare systems increase rapidly, sponsors must break down the silos that exist between CTs and RWE. Through record linkage, sponsors may potentially reduce study costs, while minimizing the patient burden to provide insights that are critical in making informed clinical decisions.

## Data Availability

No data were used in this article.
